# First introduction of highly pathogenic H5N1 avian influenza A viruses in wild and domestic birds in Denmark, Northern Europe

**DOI:** 10.1186/1743-422X-4-43

**Published:** 2007-05-11

**Authors:** Karoline Bragstad, Poul H Jørgensen, Kurt Handberg, Anne S Hammer, Susanne Kabell, Anders Fomsgaard

**Affiliations:** 1 Laboratory for Virus Research and Development, Statens Serum Institut, Artillerivej 5, DK-2300 Copenhagen S, Denmark; 2 National Veterinary Institute, Technical University of Denmark, Hangøvej 2, DK-8200 Aarhus N, Denmark

## Abstract

**Background:**

Since 2005 highly pathogenic (HP) avian influenza A H5N1 viruses have spread from Asia to Africa and Europe infecting poultry, humans and wild birds. HP H5N1 virus was isolated in Denmark for the first time in March 2006. A total of 44 wild birds were found positive for the HP H5N1 infection. In addition, one case was reported in a backyard poultry flock.

**Results:**

Full-genome characterisation of nine isolates revealed that the Danish H5N1 viruses were highly similar to German H5N1 isolates in all genes from the same time period. The haemagglutinin gene grouped phylogenetically in H5 clade 2 subclade 2 and closest relatives besides the German isolates were isolates from Croatia in 2005, Nigeria and Niger in 2006 and isolates from Astrakhan in Russia 2006. The German and Danish isolates shared unique substitutions in the NA, PB1 and NS2 proteins.

**Conclusion:**

The first case of HP H5N1 infection of wild and domestic birds in Denmark was experienced in March 2006. This is the first full genome characterisation of HP H5N1 avian influenza A virus in the Nordic countries. The Danish viruses from this time period have their origin from the wild bird strains from Qinghai in 2005. These viruses may have been introduced to the Northern Europe through unusual migration due to the cold weather in Eastern Europe at that time.

## Background

All subtypes of influenza A are perpetuated in wild aquatic birds and thereby these birds serve as a reservoir of influenza A. Avian influenza viruses (AIVs) are believed to be in evolutionary stasis in its natural hosts of wild birds where the virus and the host tolerate each other [1]. AIVs are characterised as low pathogenic (LP) or high pathogenic (HP), depending on their ability to cause disease in chickens. LP AIV may become HP to poultry through mutations after introduction from wild birds. Until now, only AIV of subtypes H5 and H7 have become HP. There are currently recognised sixteen subtypes of haemagglutinin (HA) and nine neuraminidases (NAs) [2,3]. Only H3N2, H1N1 and H1N2 out of 144 theoretically possible subtype combinations circulate in humans. In Hong Kong in 1997, eighteen people became infected with HP AIV H5N1 and six died [4,5]. Before 1997, AIV was not expected to cross the species barrier and infect humans, although incidences of H7N7 infection had been seen in the past [6-8]. Prior to the outbreak in humans in 1997 HP H5N1 viruses had circulated in poultry in the same region and HP AIV H5N1 viruses persisted in geese and ducks [9-11]. In 2002 a HP H5N1 strain re-emerged in Hong Kong and for the first time lethal influenza virus infection was observed in wild aquatic birds [12]. After further genetic change the HP H5N1 re-emerged in poultry and humans in Hong Kong 2003 [13]. There have been sporadic human cases of HP AIV H5N1 infections since then and until the start of 2007 more than 290 people have become infected and more than 170 have died from the infection. Apparently the virus has become endemic in poultry in Asia and has now spread to Africa and Europe.  In contrast to previous believes wild migratory birds might play some role in the transmission of HP AIV. In 2005, thousands of migratory waterfowl in Qinghai Lake, western China, became infected with HP AIV H5N1 and this might have contributed to the spread of HP H5N1 to Europe and Africa in 2005 [14].

In Denmark, the first case of HP AIV H5N1 in wild birds was identified in March 2006 [15], after the virus had been discovered in many parts of Europe in 2005/2006, including neighbour countries to Denmark like Germany [16] and Sweden. HP AIV had never been detected in Denmark before 2006; however, a case of LP H5N2 infection in quarantined ostriches was observed in 1996 [17] and LP H5N7 virus was discovered in mallards bred for restocking of game in 2003 [18,19]. Increased surveillance since then has elucidated the presence of both LP H5N2 and H5N3 viruses in game stocks. This paper characterises the genomes of the first cases of HP H5N1 viruses that infected wild and domestic birds in Denmark in 2006.

## Results and discussion

In 2006, 1,381 dead wild birds were brought to the Danish National Veterinary Institute. Of these, 44 were positive for HP H5N1 distributed as follows; three whooper swans, four mute swans, one greylag goose, 26 tufted ducks, six common buzzards, one rough-legged buzzard, one peregrine falcon, one great crested grebe and one magpie. In addition, four birds in a hobby poultry flock tested positive for HP H5N1: one peacock, one fowl and two muscovy ducks. The detected HP H5N1 cases were mainly found in the Baltic Sea area of Denmark.

**Figure 1:**
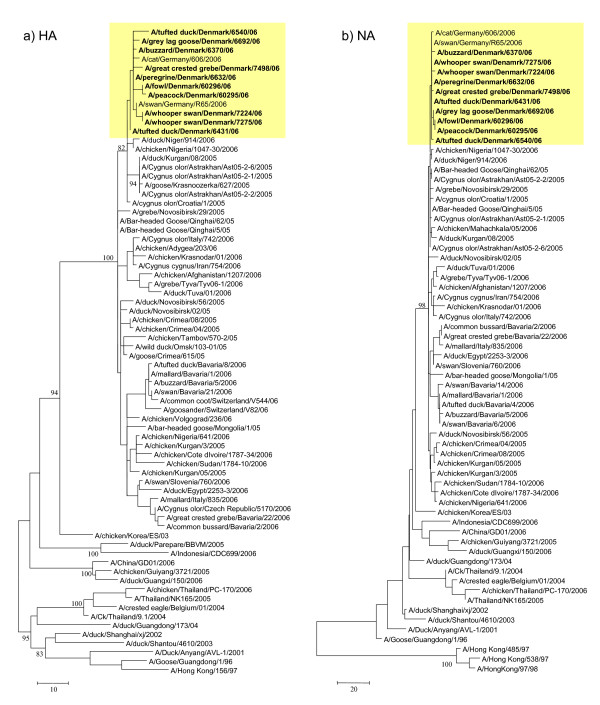
**Evolutionary relationship of HA and NA genes of Danish HP H5N1 AIVs compared to other past and present H5N1 viruses**. The nucleotide coding region trees were generated by maximum parsimony with heuristic random branch swapping search (neighbor joining and maximum likelihood analyses revealed similar tree topologies). Bootstrap values of 1000 resamplings in per cent (>70%) are indicated at key nodes. The clade with Danish isolates is marked in yellow. Figure 1 a) and b) demonstrate the evolutionary relationship of the HA and NA genes, respectively. Trees for HA and NA includes A/fowl/Denmark/60296/06, this virus was not full genome amplified and therefore not present in trees representing genes coding for the internal proteins. Trees are midpoint rooted for means of clarity.

**Figure 2:**
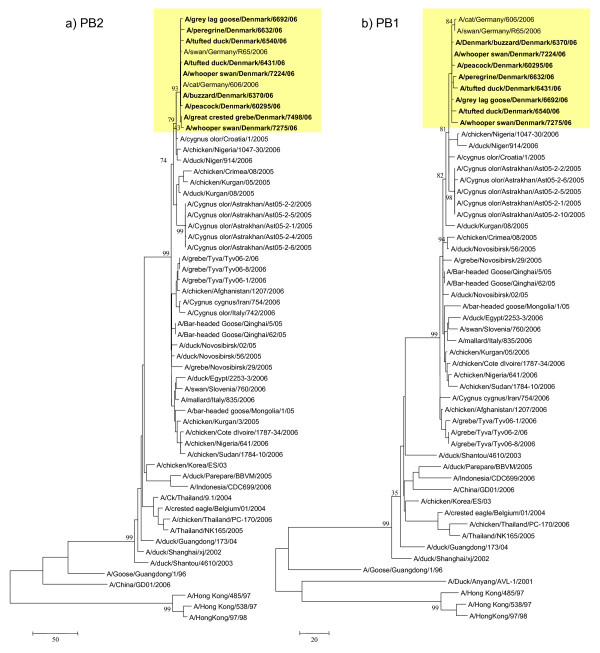
**Evolutionary relationship of PB2 and PB1 genes of Danish HP H5N1 AIVs compared to other past and present H5N1 viruses**. The nucleotide coding region trees were generated by maximum parsimony with heuristic random branch swapping search (neighbor joining and maximum likelihood analyses revealed similar tree topologies). Bootstrap values of 1000 resamplings in per cent (>70%) are indicated at key nodes. The clade with Danish isolates is marked in yellow. Figure 2 a) and b) demonstrate the evolutionary relationship of the PB2 and PB1 genes, respectively. Trees are midpoint rooted for means of clarity.

**Figure 3:**
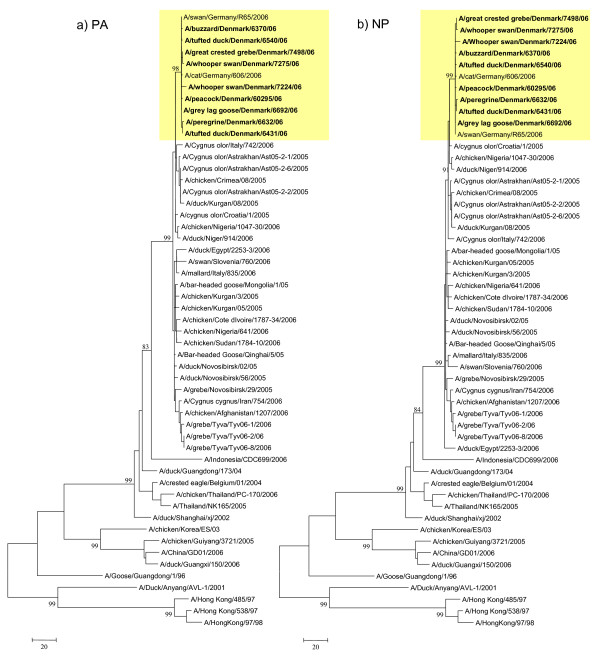
**Evolutionary relationship of PA and NP genes of Danish HP H5N1 AIVs compared to other past and present H5N1 viruses**. The nucleotide coding region trees were generated by maximum parsimony with heuristic random branch swapping search (neighbor joining and maximum likelihood analyses revealed similar tree topologies). Bootstrap values of 1000 resamplings in per cent (>70%) are indicated at key nodes. The clade with Danish isolates is marked in yellow. Figure 3 a) and b) demonstrate the evolutionary relationship of the PA and NP genes, respectively. Trees are midpoint rooted for means of clarity.

**Figure 4:**
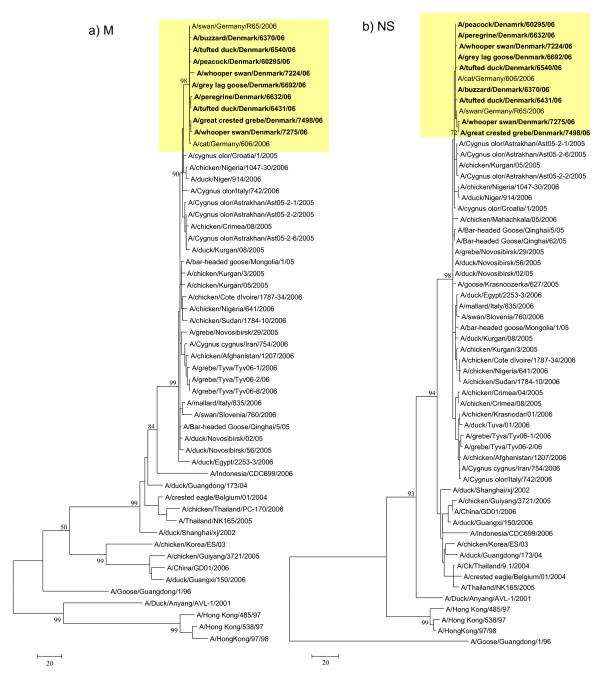
**Evolutionary relationship of M and NS genes of Danish HP H5N1 AIVs compared to other past and present H5N1 viruses**. The nucleotide coding region trees were generated by maximum parsimony with heuristic random branch swapping search (neighbor joining and maximum likelihood analyses revealed similar tree topologies). Bootstrap values of 1000 resamplings in per cent (>70%) are indicated at key nodes. The clade with Danish isolates is marked in yellow. Figure 4 a) and b) demonstrate the evolutionary relationship of the M and NS genes, respectively. Trees are midpoint rooted for means of clarity.

The Danish and German isolates group together phylogenetically, forming a monophyletic cluster in all genes indicating that these isolates have evolved from the same origin (Fig. 1, 2, 3 and 4). Genes from the index case, A/buzzard/Denmark/6370/06(H5N1), had a 99.8–100% nucleotide sequence identity to the German isolates. The Danish isolates were further located in the defined phylogenetic H5 clade 2 subclade 2 as the majority of the European strains. Besides the German viruses, the Danish viruses were highly similar to viruses from Croatia, Nigeria, Niger and also Astrakhan in Russia (Fig. 1, 2, 3 and 4). Characterisation of the German isolates by Weber *et. al*., [16] indicated a closer relation to the Russian Astrakhan isolates than shown here. We show that the Russian isolates are closely related to the German and Danish isolates but form a separate monophyletic cluster with high bootstrap values (94%). This topology was seen for neighbor joining, maximum parsimony and maximum likelihood methods. We see a difference probably because we were able to include more contemporary European isolates in the analysis. The Danish viruses from this time period have their origin in the Qinghai wild bird strains from 2005. The same topology was observed for all eight genes. The Qinghai Lake viruses could be traced to viruses isolated from wild birds at the Poyang Lake 1,700 km away. It has therefore been suggested that these viruses have been spread by infected migratory birds [20]. It is most likely that the introduction of HP H5N1 AIV to Denmark is caused by an unusual migration of infected birds due to the harsh cold weather in Eastern Europe as suggested also by others [16,21]. This theory could be further examined when more AIV sequences are made available.

The two amino acid residues in HA, I83 and N252 and NA-R110, assumed to be unique for migratory birds at Qinghai and Poyang Lakes [20] were also present in the Danish isolates. The Danish strains possessed a multi-basic HA0 amino acid cleavage sequence, PQGERRRKKR/GLF, characteristic for strains highly pathogenic to poultry. This cleavage sequence is present in the majority of the European HP H5N1 strains from the same time period and for the Qinghai viruses that infected wild birds in 2005. The NA protein of the Danish isolates were characterised by a 20 amino acid deletion at positions 49 to 68 in the stalk region of NA. This deletion has been observed in many recent HP H5N1 isolates [16,22,23] and is suggested to be adaptation for efficient replication in chickens [24].

One genetic indicator for high pathogenicity in mice and adaptation for efficient replication in humans is the E627K substitution in the PB2 protein [25,26]. All Danish isolates possessed the PB2-K627 amino acid. In addition the amino acid E92 in the NS1 protein observed for the Danish isolates is associated with increased virulence in pigs [27,28]. The isolates possessed a ESKV amino acid sequence at the NS1 C-terminal end. This sequence was also observed for all other isolates in the clade 2 subclade 2 cluster. This C- terminal region might be involved in binding to PDZ domains on proteins involved in host cellular signalling pathways [29]. A PDZ-binding domain has been present in all virulent H5N1 viruses isolated from humans [30]. Therefore a functional PDZ-binding domain is suggested to correlate with human virulence. Although suggested that the majority of avian strains possess an ESEV PDZ domain motif, only 11 out of 60 sequences included in this analysis did possess this motif. Human viruses generally have a different motif, RSKV, that do not bind PDZ [30,31]. It is unknown what effect the ESKV motif has on influenza virus interactions; however, this motif has been shown to be a potent type 1 PDZ-binding domain in other systems [32,33].  Other substitutions that might influence on pathogenicity are the L13P and S678N in the PB1 protein and N319K in NP [34]. Only L13P in the PB1 protein was present in the Danish isolates. One substitution in PB1 that might be involved in human adaptation, N375S, found in the three recent human pandemic strains [35], was absent in our HP AIV H5N1 isolates.

Species-specific residues in the different proteins after the list of Shaw *et al*., [36] were all avian-like with the exception of NP-I33 and M2-V27 which are human specific residues. The HA molecules of the Danish strains have preferred receptor specificity for the avian α-(2,3) linkage to galactose indicated by the Q226 and G228 amino acid residues in HA [37-39].

The mean differences between the HAs from the Danish isolates were 4.7 nucleotides or 1.9 amino acids. The viruses isolated from domestic birds, a peacock and a fowl, varied from the wild bird isolates in the HA protein with the substitutions R162I and I232V (166 and 236 in H3 numbering). Position 162 might influence on the antigenic sites due to its location close to residues directly involved in these sites. The swan isolates varied from the other isolates with the substitution V57I in HA (66 in H3 numbering). None of these substitutions were found in other sequences included in the dataset for HA (n = 60). All Danish isolates, except for A/tufted duck/Denmark/6431/06, were characterised by the substitution D387N (386 in H3 numbering) also found in the German isolates and the human isolate A/Hong Kong/485/97. The sequence variation observed between the wild bird and domestic bird isolates could indicate adaptation to poultry or positions important for egg growth (samples from domestic birds were not propagated in eggs). Further analysis on HP H5N1 isolates from domestic birds would clarify any difference between wild birds and poultry isolates.  The A/gray lag goose/Denmark/6692/06 isolate possessed a I398M substitution in the NA protein as did the two domestic bird isolates. This substitution was also found in human infectious HP H5N1 strains from 1997 and 1998. It could be interesting to further investigate if this substitution is involved in human adaptation. NA-G316D was unique for the Danish and the German isolates. Position 316 is part of the N2 antigenic site C (336 in N2 numbering).

A PB1-K531R substitution characterised the Danish isolates (except A/whooper swan/Denmark/7275/06 (PB2-M483)) and isolates from Germany. The Danish isolates possessed the substitutions NP-V270I and NS1-V194I. The NS1 proteins were also characterised by a five amino acid deletion at positions 80 to 84 as also observed for H5N1 viruses of the Z+, Z, Y, A, B and C genotypes [40]. The NS2 genes possessed the G63E substitution also found in the German isolates.

It has been suggested that an additional glycosylation site at position 158 (H3 numbering) located on the globular head improves elution of virus from erythrocytes [41] and compensates for the weak enzymatic activity of NA molecules posed by deletions in the stalk region [24]. The Danish H5 isolates possessed four potential N-linked glycosylation sites in HA1 at positions 11, 23, 165, and 286 (21, 33, 169 and 289 in H3 numbering) with predicted threshold values above 0.5 (not shown). Position 193 (198 in H3 numbering) was also suggested; however, proline occurs just after the asparagine residue and is therefore highly unlikely glycosylated due to conformational constraints. Three N-linked glycosylated sequons were predicted for N1 at positions 68, 126 and 215 (88, 146 and 235 when including the 20 amino acid deletion in the stalk region).

Due to the potential risk of infection to humans in close contact with infected birds, we looked specifically for genetic indications for influenza inhibitory drug resistance but could not identify amino acid substitutions previously demonstrated to infer resistance to neither neuraminidase inhibitory drugs like oseltamivir nor matrix protein inhibitory drugs like amantadine [42-45].

## Conclusion

HP H5N1 infection in both wild and domestic birds was observed for the first time in Denmark in March 2006. The viruses identified in Denmark were highly similar to viruses from cases in Germany from the same time period and have their origin in the Qinghai-like viruses. The Danish and German isolates share unique amino acids in the NA (G336D), PB1 (K531R) and NS2 (G63E) proteins.

We emphasise the importance of full genome sequencing of emerging new viruses to elucidate the migration of emerging new strains and the evolution of viruses over time. Knowledge about the circulating genomes might increase the understanding about the spread and mechanisms of virulence and pathogenicity of H5N1 viruses to poultry and humans.

## Methods

### Detection of avian influenza A virus in trachea swab samples from dead wild birds

The dead wild birds were received at the Danish National Veterinary Institute from several locations in Denmark, in the spring of 2006. Trachea swabs were transferred to 10 ml centrifuges tubes containing 2.5 ml PBS with 2,000 i.e. penicillin/ml, 2 mg/ml streptomycin, 5 % foetal bovine calf serum and 0.1 % Phenol Red, pH: 7.3. The tubes were shaken for 30 minutes and the supernatant was collected after centrifugation.

### Virus isolation in SPF eggs

Cultivation of virus was done according to EU directive 2005/94/EEC. Briefly, swab elutes including antibiotics were inoculated into the allantoic cavity of 8 to 10-day-old specific pathogen-free (SPF) chicken embryos. The eggs were incubated in a humidified atmosphere at 37°C and candled daily. Allantoic fluid harvested from eggs with dead embryos and from eggs incubated for six days was examined for haemagglutinating activity. At least two serial blind passages were made.

### Initial RNA extraction and partial RT-PCR

Total RNA was prepared from supernatants by the RNeasy Mini Kit (QIAGEN, Hilden, Germany). A modified version of the manufacturer's protocol was followed by mixing 300 μl of lysis buffer (RTL) containing 6 μl β-mercaptoethanol (Sigma) with 400 μl eluate from tracheal swabs and left for at least 10 minutes at room temperature. If the mixture was cloudy, the supernatant was transferred to a new tube after a short centrifugation. After the addition of 700 μml 70% ethanol and mixing, the liquid was applied to a spin-column in two steps. Washing and elution was done according to the manufacturers instructions. The elution volume was 50 μml.

An RT-PCR was carried out according to the manufacturer's instructions for QIAGEN OneStep RT-PCR Kit (QIAGEN, Hilden, Germany). The reaction was carried out by use of the programme cycle 1: 30 min at 50°C (RT-reaction); cycle 2: 94°C for 60 sec.; cycle 3–12: 94°C for 30 sec., 58°C for 60 sec. and 72°C for 60 sec.; cycle 26–37: 94°C for 30 sec., 58°C for 60 sec. and 72°C for 65 sec. plus an extra 5 sec. per cycle; cycle 38: 72°C for 7 minutes on a thermocycler (PTC-200, MJ Research or T3 Thermocycler, Biometra). The presence of PCR products were confirmed by agarose electrophoresis, using a 1.5% SeaKem GTG (FMC) agarose and 0.1 μml/ml ethidium bromide. RT-PCR primers were matrix forward primer FB-AI-M52C: 5'-CTT CTA ACC GAG GTC GAA ACG-3', matrix reverse primer FB-AI-M253R: 5'-AGG GCA TTT TGG ACA AAK CGT CTA-3', H5 forward primer KHA-1 5'-CCT CCA GAR TAT GCM TAY AAA ATT GTC-3' and H5 reverse primer KHA-3: 5'-TAC CAA CCG TCT ACC ATK CCY TG-3'. The PCR fragments were extracted from the agarose gels by Qiaquick Gel Extraction Kit (QIAGEN, Hilden, Germany). Sequencing was performed by DNA Technology a/s, Aarhus, Denmark. A sample was only considered positive for H5 if both the matrix and the H5 RT-PCR were positive and in addition the sequences gave a positive Blast search for H5 in GenBank.

### Full-genome RT-PCR and sequencing of nine isolates

Viral RNA was extracted from 140 μl of egg-cultivated, virus suspensions in a biosafety level 3 laboratory by QIAamp Viral RNA Mini Kit (QIAGEN) as described in the kit protocol. RNA from two domesticated birds, A/peacock/Denmark/60295/06 and A/fowl/Denmark/60296/06, were extracted directly from oropharyngeal swabs. The different gene segments were amplified by OneStep RT-PCR Kit (QIAGEN) as previously described [19], a two minute elongation time for all genes were applied. The primers for RT-PCR were segment specific but subtype universal targeting the highly conserved noncoding RNA regions at the 5'- and 3'-end of each segment [46]. PCR products were purified with the GFX™ PCR DNA and Gel Band Purification Kit (Amersham Biosciences, Piscataway, USA) prior to sequencing. Purified PCR products were sequenced directly. The RT-PCR and sequencing primer sequences are available upon request. The sequencing reaction was performed by ABI PRISM BigDye Terminators v3.1 Cycle Sequencing Kit (Applied Biosystems, Foster City, California, USA) as described previously [18]. The development of the sequences was performed on an automatic ABI PRISM 3130 genetic analyzer (Applied Biosystems) with 80 cm capillaries. Consensus sequences were generated in SeqScape Software v2.5 (Applied Biosystems). Sequence assembly, multiple alignment and alignment trimming were performed with the BioEdit software v.7.0.5 [47]. Distance based neighbor joining and character based maximum parsimony phylogenetic trees were generated using the Molecular Evolutionary Genetics Analysis (MEGA) software v.3.1 [48]. Maximum likelihood phylogenetic trees were generated by Phylogenetic Analysis Using Parsimony (PAUP 4.0) Software (Sinauer Associates, Inc.) [49] applying the HKY85 nucleotide model, allowing transitions and transversions to occur at different rates. Included in the trees with the nine Danish HP H5N1 full-genome sequences are H5N1 strains from Europe and representative human and avian H5N1 full genomes from the rest of the world published in the Influenza Virus Resource at NCBI [50].

Amino acid residues in HA are numbered by H5 numbering if not stated otherwise.

Potential N-linked glycosylation sites were predicted using nine artificial neural networks with the NetNGlyc 1.0 Server [51]. A threshold value of average potential score >0.5 was set to predict glycosylated sites.

### Nucleotide sequence accession numbers

Nucleotide sequences from Danish H5N1 virus isolates included in this study have been submitted to GenBank with the following accession numbers; HA: EF523687–EF523696, NA: EF523697–EF523705, PB2: EF523706–EF523714, PB1: EF523715–EF523723, PA: EF523724–EF523732, NP: EF523733–EF523741, M: EF523742–EF523750, NS: EF523751–EF523769.

## Competing interests

The author(s) declare that they have no competing interests.

## Authors' contributions

KB carried out the full length RT-PCR analysis, sequencing and sequence analysis, alignments, full genome characterisations, phylogenetics and drafted and wrote the manuscript. PHJ purified clinical samples, cultivated, isolated and identified viruses, carried out initial partial RT-PCR diagnostics and epidemiology analysis. PHJ also contributed to and revised the manuscript. KH cultivated virus, extracted viral RNA, carried out initial partial RT-PCR and sequencing and contributed to and revised the manuscript. ASH and SK carried out post mortem pathology, sampling, determination of species and registered and reported findings. AF contributed to conception and design of the study and interpretation of the data. Further AF critically revised the manuscript and gave the final approval for publication.

## References

[B1] Gorman OT, Bean WJ, Webster RG (1992). Evolutionary processes in influenza viruses: divergence, rapid evolution, and stasis. Curr Top Microbiol Immunol.

[B2] Fouchier RA, Munster V, Wallensten A, Bestebroer TM, Herfst S, Smith D, Rimmelzwaan GF, Olsen B, Osterhaus AD (2005). Characterization of a novel influenza a virus hemagglutinin subtype (H16) obtained from black-headed gulls. J Virol.

[B3] Webster RG, Bean WJ, Gorman OT, Chambers TM, Kawaoka Y (1992). Evolution and ecology of influenza A viruses. Microbiol Rev.

[B4] Claas EC, de Jong JC, van Beek R, Rimmelzwaan GF, Osterhaus AD (1998). Human influenza virus A/HongKong/156/97 (H5N1) infection. Vaccine.

[B5] Claas EC, Osterhaus AD, van Beek R, de Jong JC, Rimmelzwaan GF, Senne DA, Krauss S, Shortridge KF, Webster RG (1998). Human influenza A H5N1 virus related to a highly pathogenic avian influenza virus. Lancet.

[B6] Banks J, Speidel E, Alexander DJ (1998). Characterisation of an avian influenza A virus isolated from a human--is an intermediate host necessary for the emergence of pandemic influenza viruses?. Arch Virol.

[B7] Kurtz J, Manvell RJ, Banks J (1996). Avian influenza virus isolated from a woman with conjunctivitis. Lancet.

[B8] Webster RG, Geraci J, Petursson G, Skirnisson K (1981). Conjunctivitis in human beings caused by influenza A virus of seals. N Engl J Med.

[B9] Cauthen AN, Swayne DE, Schultz-Cherry S, Perdue ML, Suarez DL (2000). Continued circulation in China of highly pathogenic avian influenza viruses encoding the hemagglutinin gene associated with the 1997 H5N1 outbreak in poultry and humans. J Virol.

[B10] Webster RG, Guan Y, Peiris M, Walker D, Krauss S, Zhou NN, Govorkova EA, Ellis TM, Dyrting KC, Sit T, Perez DR, Shortridge KF (2002). Characterization of H5N1 influenza viruses that continue to circulate in geese in southeastern China. J Virol.

[B11] Tumpey TM, Suarez DL, Perkins LE, Senne DA, Lee JG, Lee YJ, Mo IP, Sung HW, Swayne DE (2002). Characterization of a highly pathogenic H5N1 avian influenza A virus isolated from duck meat. J Virol.

[B12] Sturm-Ramirez KM, Ellis T, Bousfield B, Bissett L, Dyrting K, Rehg JE, Poon L, Guan Y, Peiris M, Webster RG (2004). Reemerging H5N1 influenza viruses in Hong Kong in 2002 are highly pathogenic to ducks. J Virol.

[B13] Peiris JSM, Yu WC, Leung CW, Cheung CY, Ng WF, Nicholls JM, Ng TK, Chan KH, Lai ST, Lim WL (2004). Re-emergence of fatal human influenza A subtype H5N1 disease. The Lancet.

[B14] Chen H, Smith GJ, Zhang SY, Qin K, Wang J, Li KS, Webster RG, Peiris JS, Guan Y (2005). Avian flu: H5N1 virus outbreak in migratory waterfowl. Nature.

[B15] Molbak K, Trykker H, Mellergaard S, Glismann S (2006). Avian influenza in Denmark, March-June 2006: public health aspects. Euro Surveill.

[B16] Weber S, Harder T, Starick E, Beer M, Werner O, Hoffmann B, Mettenleiter TC, Mundt E (2007). Molecular analysis of highly pathogenic avian influenza virus of subtype H5N1 isolated from wild birds and mammals in northern Germany. J Gen Virol.

[B17] Jørgensen PH, Nielsen OL, Hansen CH, Manvell RJ, Banks J, Alexander DJ (1998). Isolation of Influenza A virus, subtype H5N2, and avian paramyxovirus type 1 from a flock of ostriches in Europe. Avian Pathology.

[B18] Bragstad K, Jorgensen PH, Handberg KJ, Mellergaard S, Corbet S, Fomsgaard A (2005). New avian influenza A virus subtype combination H5N7 identified in Danish mallard ducks. Virus Res.

[B19] Bragstad K, Jorgensen PH, Handberg KJ, Fomsgaard A (2006). Genome characterisation of the newly discovered avian influenza A H5N7 virus subtype combination. Arch Virol.

[B20] Chen H, Smith GJD, Li KS, Wang J, Fan XH, Rayner JM, Vijaykrishna D, Zhang JX, Zhang LJ, Guo CT, Cheung CL, Xu KM, Duan L, Huang K, Qin K, Leung YHC, Wu WL, Lu HR, Chen Y, Xia NS, Naipospos TSP, Yuen KY, Hassan SS, Bahri S, Nguyen TD, Webster RG, Peiris JSM, Guan Y (2006). Establishment of multiple sublineages of H5N1 influenza virus in Asia: Implications for pandemic control. PNAS.

[B21] Kilpatrick AM, Chmura AA, Gibbons DW, Fleischer RC, Marra PP, Daszak P (2006). From the Cover: Predicting the global spread of H5N1 avian influenza. PNAS.

[B22] Viseshakul N, Thanawongnuwech R, Amonsin A, Suradhat S, Payungporn S, Keawchareon J, Oraveerakul K, Wongyanin P, Plitkul S, Theamboonlers A, Poovorawan Y (2004). The genome sequence analysis of H5N1 avian influenza A virus isolated from the outbreak among poultry populations in Thailand. Virology.

[B23] Ducatez MF, Olinger CM, Owoade AA, De Landtsheer S, Ammerlaan W, Niesters HG, Osterhaus AD, Fouchier RA, Muller CP (2006). Avian flu: multiple introductions of H5N1 in Nigeria. Nature.

[B24] Matrosovich M, Zhou N, Kawaoka Y, Webster R (1999). The surface glycoproteins of H5 influenza viruses isolated from humans, chickens, and wild aquatic birds have distinguishable properties. J Virol.

[B25] Shinya K, Hamm S, Hatta M, Ito H, Ito T, Kawaoka Y (2004). PB2 amino acid at position 627 affects replicative efficiency, but not cell tropism, of Hong Kong H5N1 influenza A viruses in mice. Virology.

[B26] Subbarao EK, London W, Murphy BR (1993). A single amino acid in the PB2 gene of influenza A virus is a determinant of host range. J Virol.

[B27] Seo SH, Hoffmann E, Webster RG (2002). Lethal H5N1 influenza viruses escape host anti-viral cytokine responses. Nat Med.

[B28] Seo SH, Hoffmann E, Webster RG (2004). The NS1 gene of H5N1 influenza viruses circumvents the host anti-viral cytokine responses. Virus Research.

[B29] Fanning AS, Anderson JM (1999). PDZ domains: fundamental building blocks in the organization of protein complexes at the plasma membrane. J Clin Invest.

[B30] Obenauer JC, Denson J, Mehta PK, Su X, Mukatira S, Finkelstein DB, Xu X, Wang J, Ma J, Fan Y, Rakestraw KM, Webster RG, Hoffmann E, Krauss S, Zheng J, Zhang Z, Naeve CW (2006). Large-scale sequence analysis of avian influenza isolates. Science.

[B31] Krug RM (2006). Virology. Clues to the virulence of H5N1 viruses in humans. Science.

[B32] Gardner LA, Naren AP, Bahouth SW (2007). Assembly of an SAP97-AKAP79-cAMP-dependent Protein Kinase Scaffold at the Type 1 PSD-95/DLG/ZO1 Motif of the Human beta1-Adrenergic Receptor Generates a Receptosome Involved in Receptor Recycling and Networking. J Biol Chem.

[B33] He J, Bellini M, Xu J, Castleberry AM, Hall RA (2004). Interaction with cystic fibrosis transmembrane conductance regulator-associated ligand (CAL) inhibits beta1-adrenergic receptor surface expression. J Biol Chem.

[B34] Gabriel G, Dauber B, Wolff T, Planz O, Klenk HD, Stech J (2005). The viral polymerase mediates adaptation of an avian influenza virus to a mammalian host. Proc Natl Acad Sci U S A.

[B35] Garcia-Sastre A, Whitley RJ (2006). Lessons learned from reconstructing the 1918 influenza pandemic. J Infect Dis.

[B36] Shaw M, Cooper L, Xu X, Thompson W, Krauss S, Guan Y, Zhou N, Klimov A, Cox N, Webster R, Lim W, Shortridge K, Subbarao K (2002). Molecular changes associated with the transmission of avian influenza a H5N1 and H9N2 viruses to humans. J Med Virol.

[B37] Matrosovich MN, Gambaryan AS, Teneberg S, Piskarev VE, Yamnikova SS, Lvov DK, Robertson JS, Karlsson KA (1997). Avian influenza A viruses differ from human viruses by recognition of sialyloligosaccharides and gangliosides and by a higher conservation of the HA receptor-binding site. Virology.

[B38] Matrosovich MN, Matrosovich TY, Gray T, Roberts NA, Klenk HD (2004). Human and avian influenza viruses target different cell types in cultures of human airway epithelium. Proc Natl Acad Sci U S A.

[B39] Suzuki Y, Ito T, Suzuki T, Holland RE, Chambers TM, Kiso M, Ishida H, Kawaoka Y (2000). Sialic acid species as a determinant of the host range of influenza A viruses. J Virol.

[B40] Guan Y, Poon LL, Cheung CY, Ellis TM, Lim W, Lipatov AS, Chan KH, Sturm-Ramirez KM, Cheung CL, Leung YH, Yuen KY, Webster RG, Peiris JS (2004). H5N1 influenza: a protean pandemic threat. Proc Natl Acad Sci U S A.

[B41] Bender C, Hall H, Huang J, Klimov A, Cox N, Hay A, Gregory V, Cameron K, Lim W, Subbarao K (1999). Characterization of the surface proteins of influenza A (H5N1) viruses isolated from humans in 1997-1998. Virology.

[B42] Hay AJ, Zambon MC, Wolstenholme AJ, Skehel JJ, Smith MH (1986). Molecular basis of resistance of influenza A viruses to amantadine. J Antimicrob Chemother.

[B43] Suzuki H, Saito R, Masuda H, Oshitani H, Sato M, Sato I (2003). Emergence of amantadine-resistant influenza A viruses: epidemiological study. J Infect Chemother.

[B44] Tisdale M (2000). Monitoring of viral susceptibility: new challenges with the development of influenza NA inhibitors. Rev Med Virol.

[B45] Ward P, Small I, Smith J, Suter P, Dutkowski R (2005). Oseltamivir (Tamiflu) and its potential for use in the event of an influenza pandemic. J Antimicrob Chemother.

[B46] Hoffmann E, Stech J, Guan Y, Webster RG, Perez DR (2001). Universal primer set for the full-length amplification of all influenza A viruses. Arch Virol.

[B47] Hall TA (1999). BioEdit: a user-friendly biological sequence alignment editor and analysis 
  program for Windows 95/98/NT. Nucl Acids Symp Ser.

[B48] Kumar S, Tamura K, Nei M (2004). Integrated Software for Molecular Evolutionary Genetics Analysis and Sequence Alignment. Briefings in Bioinformatics.

[B49] Swafford DL (2003). Phylogenetic Analysis Using Parsimony, PAUP.

[B50] (2004). Influenza Virus Resource. http://www.ncbi.nlm.nih.gov/genomes/FLU/.

[B51] Gupta R, Jung E, Brunak S (2004). Prediction of N-glycosylation sites in human proteins. http://www.cbs.dtu.dk/services/NetNGlyc/.

